# Additive Effects of Prior Knowledge and Predictive Visual Information in Improving Continuous Tracking Performance

**DOI:** 10.5334/joc.130

**Published:** 2020-10-13

**Authors:** Laura Broeker, Harald Ewolds, Rita F. de Oliveira, Stefan Künzell, Markus Raab

**Affiliations:** 1 German Sport University Cologne, Institute of Psychology, Cologne, DE; 2 Augsburg University, Institute of Sports Science, Augsburg, DE; 3 London South Bank University, School of Applied Sciences, London, UK

**Keywords:** Action and perception, Auditory processing, Implicit learning, Visual perception

## Abstract

Visual information and prior knowledge represent two different sources of predictability for tasks which each have been reported to have a beneficial effect on dual-task performance. What if the two were combined? Adding multiple sources of predictability might, on the one hand, lead to additive, beneficial effects on dual-tasking. On the other hand, it is conceivable that multiple sources of predictability do not increase dual-task performance further, as they complicate performance due to having to process information from multiple sources. In this study, we combined two sources of predictability, predictive visual information and prior knowledge (implicit learning and explicit learning) in a dual-task setup. 22 participants performed a continuous tracking task together with an auditory reaction time task over three days. The middle segment of the tracking task was repeating to promote motor learning, but only half of the participants was informed about this. After the practice blocks (day 3), we provided participants with predictive visual information about the tracking path to test whether visual information would add to beneficial effects of prior knowledge (additive effects of predictability). Results show that both predictive visual information and prior knowledge improved dual-task performance, presented simultaneously or in absence of each other. These results show that processing of information relevant for enhancement of task performance is unhindered by dual-task demands.

Humans continually predict events in their environment, updating representations of the external world as they encounter irregularities (Friston, 2010; Wolpert et al., 2003). This constant adaptation likely happens without awareness or the need of attentional resources (Whittlesea, 2004). A reduction, or adaptive use, of attentional resources is of major relevance to researchers interested in optimizing dual-task performance since the finding that dual-task performance is inferior to single-task performance is often attributed to a limited pool of resources (Wickens, 2008). While making tasks more predictable has been generally beneficial for reducing resource requirements, results have not always been straightforward for dual-task improvement, because more predictability does not necessarily lead to increased benefits or interference reduction (de Oliveira et al., 2014). A possible reason for these findings is that predictability not only reduces resource requirements, it also affects resource allocation policy and potentially unequal weighting of tasks (Broeker et al., 2018). This might be complicated further when multiple sources of predictability are available.

In the current paper we provide prior knowledge and predictive visual information as two sources of predictability in a continuous tracking task, with the aim to investigate how multiple sources of predictability interact in improving dual-task performance. In the literature, predictability has been argued to come from the individual through prior knowledge or from information available in the environment (Gentsch et al., 2016; Körding & Wolpert, 2006). We considered two possible outcomes: a) after having learnt a repeating pattern (implicitly or explicitly) participants will improve further as soon as predictive visual information is added to prior knowledge showing additive effects; b) after having learnt a repeating pattern (implicitly or explicitly) participants will no longer rely on prior knowledge as soon as predictive visual information is added and adapt to a feedforward online control strategy (see Figure 1).

**Figure 1:**
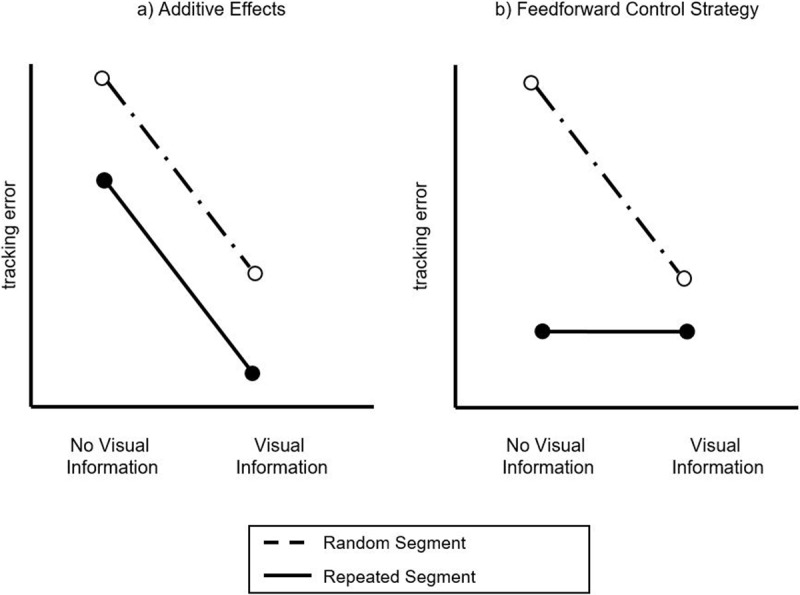
Schematic illustration of the two hypothesized effects when adding knowledge and visual information as two sources of predictability. The left half shows additive effects, meaning that participants do not only lower the tracking error on repeated vs. random segments, but improve further when visual information is added. The right half shows the alternative option of visual information winning the race for resources and determining actions based on a feedforward control strategy.

The question of whether and how different sources of information interact has been the core interest of various experimental manipulations. While some researchers found that planned actions are abandoned when information in the environment is added (e.g. Pfister et al., 2012), others found that planned actions are more influential than information in the environment (i.e. cues) for subsequent behavior (e.g. Gozli et al., 2016; Kemper et al., 2012). Again others demonstrated that previously learnt knowledge about sequences is not abandoned when information in the environment is additionally presented (Gaschler et al., 2018). The variance in experimental paradigms used (e.g. discrete response-time tasks, memory tasks, visual search tasks) however prove a clear inference of hypotheses from these studies difficult and there are no studies examining the interaction of knowledge and information in continuous tasks. This complicates a mechanistic view, as for instance proposed in race models (e.g. Schuck et al., 2012), because no specific time point where knowledge vs. information is retrieved can be determined. For our design, we trust that the use of knowledge and visual information can be independently manipulated to test the alternative hypotheses presented.

In addition, we will examine the differential effects of implicit vs. explicit knowledge. Implicit knowledge, in contrast to explicit knowledge, is generally assumed to not require attentional resources (Kal et al., 2018). This means that implicit knowledge should be superior to explicit knowledge in dual-task situations. Regarding our manipulations, the expectation is that the addition of implicit knowledge and predictive visual information yields better performance compared to explicit knowledge and predictive visual information, accepting that visual information demands significant processing itself.

## Beneficial impact of knowledge vs. information on dual-task performance

The two sources of predictability, and their potential interaction become clear when considering for instance a tennis player predicting the trajectory of an incoming ball by integrating knowledge from previously played rallies vs. the visual flight path information (Körding & Wolpert, 2004). Both sources of predictability have been independently demonstrated to have beneficial effects in single- and dual-task performance, as we will review below.

Beneficial effects of prior knowledge on task performance, mainly in the form of implicit knowledge, have been demonstrated in many serial reaction-time tasks (Nissen & Bullemer, 1987; Röttger et al., 2017) and tracking studies (Ewolds et al., 2017; Pew, 1974; Tsang & Chan, 2015). In these studies, a path-to-be-tracked or a sequence of buttons-to-be-pressed is repeated over numerous trials and thus builds up knowledge about the course of the task. Results of these experiments consistently show that performance on practiced sequences is better than on random sequences. However, participants are often unaware of the existence of sequences despite learning them, and thus the difference in performance between practiced and random trials is often taken as a measure of *implicit* learning. Implicit knowledge may have an advantage over explicit knowledge in dual-task situations since no conscious processes can interfere with a secondary task. In tracking studies, however, it has been shown that participants receiving explicit instructions about sequences are advantaged over participants learning them implicitly (Ewolds et al., 2017), possibly because explicit instructions draw attention to the regularity in the sequence and ensure that knowledge is established sooner. The resulting knowledge, however, could still become implicit, in line with traditional training approaches arguing that explicit instructions first lead to declarative knowledge about movements but often become more automatic after training. A review by Kal et al. (2018) demonstrated that there is indeed little evidence to suggest that implicit learning leads to a higher degree of automaticity than explicit learning for more complex movements. Therefore, the differences between the explicit group and the implicit group in the current study should be regarded as effects of instructions rather than absence of explicit knowledge (for the implicit group) or a clear distinction between implicit and explicit knowledge. In any case, the explicit group was means to guarantee that knowledge vs. visual information can be compared.

Beneficial effects of predictive visual information have been demonstrated in a driving simulation study by de Oliveira and Wann (2010). They showed that increasing visual information linearly improved driving performance in healthy controls, yet too much information was suboptimal for people with developmental coordination disorder. They hypothesized that in people with the disorder, too much visual information overloaded their processing system, because it cannot be immediately used for online control and interferes with concurrent performance. For example, seeing the first curve of the road ahead is helpful because it allows for concurrent planning ahead, while presenting the second and third curve may interfere with planning of the first curve (see also Raab et al., 2013). If coordination disorders share characteristics with increased processing load under dual-task requirements in healthy adults, then the results would speak against our hypothesized additive effects because too much information would be present when predictive visual information and prior knowledge were added. Related evidence comes from tracking studies applying predictive visual information. Broeker et al. (under revision) demonstrated that while dual-task tracking performance improved with 200–400 ms predictive visual information, 600–800 ms did not add to tracking improvements, so a higher amount of predictive visual information can be debilitative to tracking performance, too.

In sum, both sources of predictability are beneficial to dual-task performance but no study to date has tested whether adding prior knowledge and (an optimal amount of) predictive visual information would be notably beneficial to continuous dual-task performance. We tested dual-task performance in a tracking task with an auditory reaction time task, where participants had acquired prior knowledge about the tracking path and additionally received predictive visual information about the path. This allowed us to test potential additive effects, but also a general interaction of the two sources. If no additive effects are found, we need to examine which source of information was used more for performance optimization. Do people rely on knowledge they have learnt, disregarding visual input? Or do they rely on the newly added visual information without drawing on prior knowledge?

## Methods

### Participants

We recruited 22 naïve participants (10 female; aged between 21 and 29 years, *M* = 24.5 years, *SD* = 2.31), on university campus via mailing list and participant database. The implicit group consisted of 10 participants and the explicit group consisted of 12 participants. Initially, both groups consisted of 12 participants, but two participants were removed due to incomplete data sets. The sample size was based on Ewolds et al. (2018). Participants had normal or corrected-to-normal vision and reported no musculoskeletal or neurological disorders. Participants gave written informed consent prior to the experiment and received a small remuneration for taking part. The experiments were approved by the local ethics committee and were performed according to the Declaration of Helsinki 2008.

### Setup

Participants were seated in a dimly lit room at a viewing distance of 60 cm to a 24” computer screen (144 Hz, 1,920 × 1,080 pixel resolution). The tracking software ran on a Windows 10, 64-bit system with a GTX750 graphics card. A spring-loaded joystick (SpeedLink Dark Tornado, max. sampling rate 60 Hz) was fixed to the table perpendicular to the midpoint of the screen at a distance of 30 cm. A pedal was fixed to the floor located on which participants placed their self-reported dominant foot (f-pro USB foot switch, 9 × 5 cm). Participants wore headphones (Sennheiser HD 65TV).

### Task and Display

#### Visuomotor tracking task

Participants operated a joystick with their self-reported dominant hand to control a white cursor cross to track a red target square. The cursor cross fit exactly in the 19 × 22 pixel target square. The position on the *x* axis of the cursor was coupled to target position, only the vertical movement of the cursor was user-controlled. This was implemented to prevent participants from moving the cursor straight to the right edge of the screen to cut trials short. The tracking path on each trial was pseudo-randomly composed of three different segments (adapted from Schmidt & Wulf, 1997) according to the formula:

f(x) = {b_0} + \sum\limits_{i = 1}^6 {{a_i}\ {\rm{sin}} (i \cdot x) + {b_i}\ {\rm{cos}} (i \cdot x)}

with *a_i_* and *b_i_* being randomly generated numbers ranging from –5 to 5 and *x* being a real number in the range [0; 2π]. As different amplitudes and number of extrema (Magill, 1998) have been shown to lead to differences in performance, all randomly generated segments were balanced on length and number of extrema beforehand. This yielded a final set of 41 segments from which the three segments were selected for each trial. To avoid the anticipation of peaks, the red target followed a constant path velocity of 10.5 cm/s, and as a result, trial length varied from 25.6 to 27.9 s depending on the curve’s trajectory. Each participant received his/her own individual repeating segment, ensuring that practice effects were not due to difficulty differences between segments or between segments of the two groups (Künzell et al., 2016). The repeating segment was always placed in the middle during the practice blocks. The two random segments on each trial were chosen so that each occurred an equal number of times.

**Figure 2:**
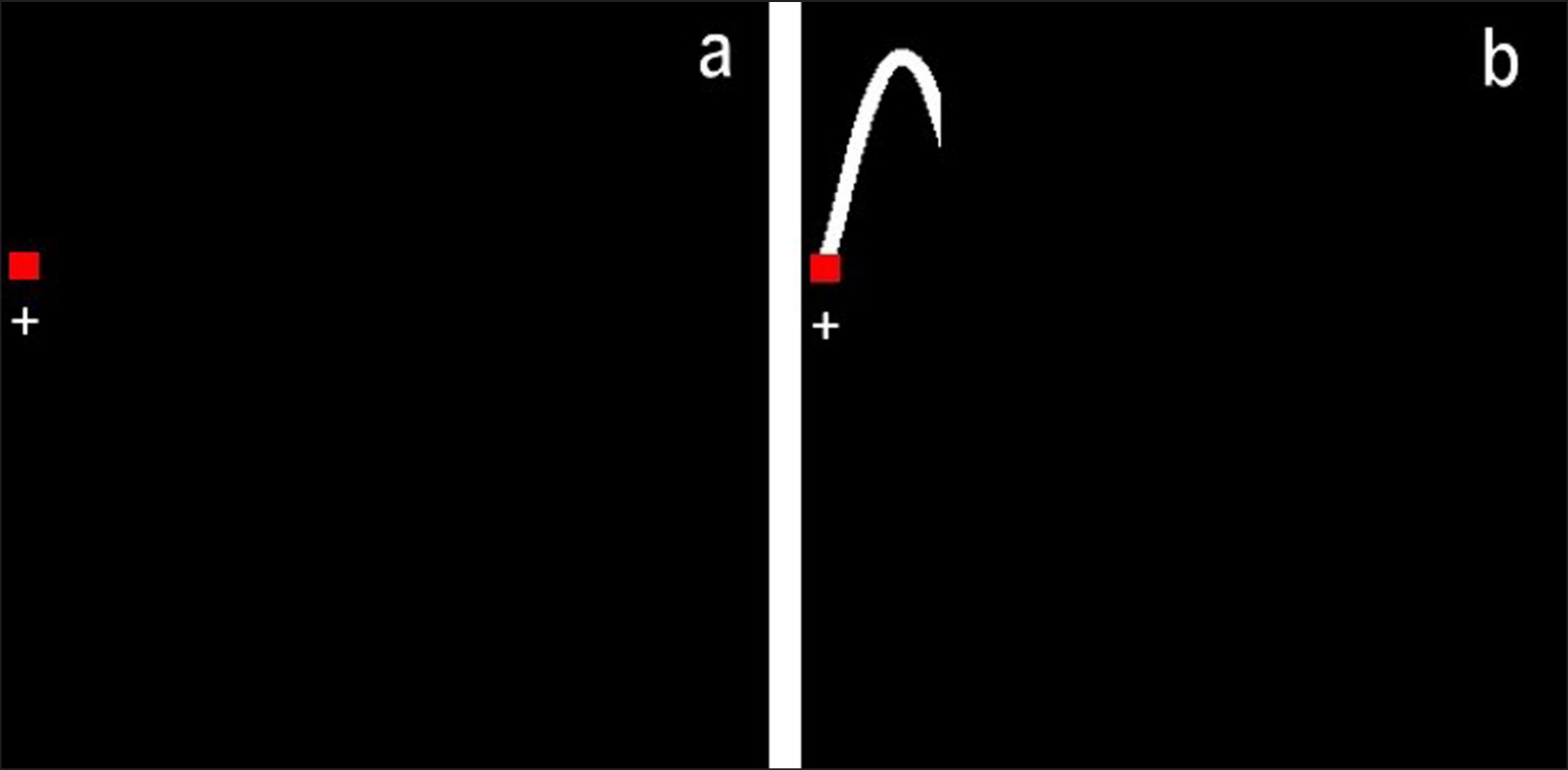
Small portions of the tracking path as displayed during the experiment. We tested the effect of displaying 400ms of the tracking path ahead (b) vs. just the target (a). Participants followed the red square and its path as accurately as possible by controlling the white cross.

In the Test Block, we added predictive visual information, so a portion of the tracking path ahead of the target was made visible (Figure 2). We chose 400 ms because Broeker et al. (under revision) have shown 400 ms to be most beneficial for dual-task (DT) cost reduction (similar to de Oliveira et al., 2014).

#### Audiomotor task

The second task was an auditory go/no-go reaction time task with high-pitched and low-pitched tones occurring randomly (1,086 Hz and 217 Hz, 75-ms duration). Participants were instructed to respond with a pedal press to high-pitched tones as fast as possible while ignoring the low-pitched tones. The number of target and distractor sounds per trial varied between 9 and 14 but all participants received the same total of sounds across the whole experiment. No tones occurred before the first 500 ms and after the last 500 ms of a trial to guarantee sufficient response time. Because average RTs for auditory discrimination in earlier DT studies were 500–950 ms (Bherer et al., 2005), the minimum gap between two sounds was 1,001 ms and responses were considered valid only when they were given within 800 ms.

### Procedure

Participants were informed that they had to practice a tracking task over several days as the study aimed at examining multitasking efficiency. While participants in the explicit group were instructed about the existence of a repeating middle segment in the tracking task, participants of the implicit group were not. All participants were instructed to follow the target square as closely as possible, to react to target tones as fast and as accurately as possible, and to put equal emphasis on both tasks. A feedback window informing participants about their tracking performance and RTs was shown after every five trials to maintain motivation (McDowd, 1986).

**Figure 3:**
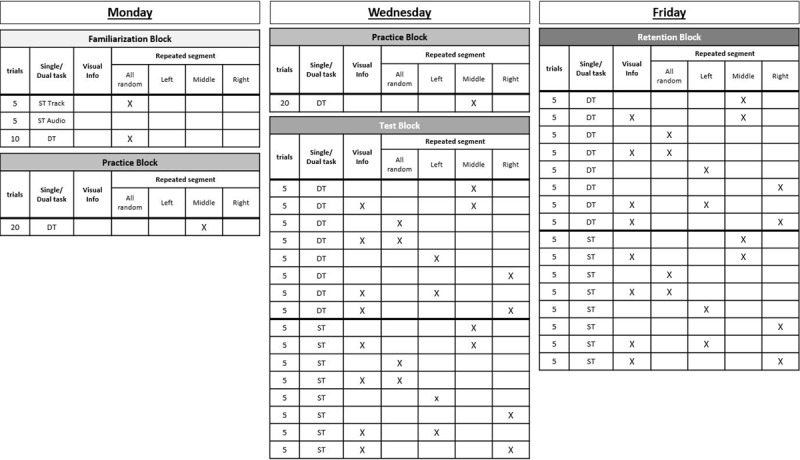
Experimental schedule over three testing days. The Familiarization and Practice blocks had 20 trials while the Test and Retention Blocks had 40 trials with a break. The 20 single- and 20 dual-task trials in the Test and Retention Block were counterbalanced across the sample. Half of the sample started with single-task trials and the other half started with dual-task trials. The third column of each block indicates whether predictive visual information was given (x = 400 ms), the fourth column indicates the presence of a repeating segment (middle = repeating segment in the middle, left or right = repeating segment that was trained in the middle was placed on the left or right, random = the repeating segment from the training blocks was replaced with a random segment).

Participants started with a familiarization block of five single-task (ST) tracking trials, then five ST auditory trials and finally two dual-task trials (without repeating segment), see Figure 3 for the experimental schedule. In the practice block the goal was for participants to learn the repeating segment, which was always placed in the middle. The number of trials followed Ewolds et al. (2017), who found implicit learning effects with a similar design. Predictive visual information was not provided during the practice block. In the Test Block, participants performed 80 trials in total: 40 dual-task and 40 single-task tracking trials, consisting of 2 predictive visual information (0 ms vs 400 ms) × 4 segment conditions (repeating in middle vs all random vs repeating left vs repeating right), each condition repeating five times. A Retention Block was performed two days later, which repeated the schedule of the Test Block. Participants were exposed to two sources of predictability for the first time during the Test Block, so a Retention Block 48 hours later was deemed adequate for sleep-dependent consolidation of motor learning to occur and provide a more accurate picture of what participants had learned (Walker & Stickgold, 2004). The usual procedure of so-called catch trials where the repeating segment is replaced by a random segment was extended with trials that positioned the repeated segment on the right or left. To the authors’ knowledge this has not been done before and gives us the opportunity to see whether position of the segment is crucial to the expression of knowledge. The explicit group was informed when the repeated segment occurred right or left, and when it was replaced by a random segment.

After finishing the experiment, participants from the implicit group were further asked to fill in a questionnaire. We used the same questionnaire as in Ewolds et al. (2017) and asked:

1. Did you notice anything special during the experiment? Yes/No; If yes, what?
2. Was there something that helped or hindered you while performing the tracking? Yes/No; If yes, what helped you?
3. Did you apply any rules? Yes/No; If yes, which?
4. Did you notice anything special concerning the path of the target? Yes/No; If yes, what?
5. The target followed a certain path. Did you notice any segments in this path? Yes/No; If yes, where?
6. There were three segments in the path, the first, the middle and at the last segment. One of these segments was always repeated. Did you notice? (7) Yes/No
  Which segment was the repeated segment? The first segment/the middle segment/the last segment

### Data Analysis

We calculated the root mean square error at 100 Hz (RMSE; 1 RMSE ≅ 0.56 cm on screen), mirroring participant’s mean deviation from the target tracking path (Schmidt & Wulf, 1997). For the tone task we recorded RTs.

Prior to the analyses we checked for outliers in the data. Participants were removed from the data set when RMSE or RTs exceeded two or more standard deviations. 2 participants from the implicit group were removed from the data set because they had only completed two testing days. To check whether learning took place for the implicit and explicit group we subjected RMSEs to a 4 × 2 × 2 mixed-measures ANOVA with factors Segment (Repeating middle vs. Repeating right vs. Repeating left vs. Random), Condition (Dual task vs. Single task) and Group (Implicit vs. Explicit), using only data without predictive visual information. We then averaged the repeating segments into a single ‘Repeating Segment’ variable to facilitate further analysis. Next, we analyzed the individual contributions of a repeating segment and predictive visual information to dual-task performance, for both RMSEs and RTs. Lastly, to test the additive contribution of both sources of predictability, we used an ANOVA including the factors Predictive Visual Information (0 ms vs. 400 ms), Segment (Repeating vs. Random), Time (Test Block vs Retention Block) and Group (Implicit vs. Explicit). An additive effect would be given if we can show main effects of Segment and Visual predictive information, and a non-significant interaction between them (APA, n.d.). This means that the difference between random and repeating segments should be unconditional upon visual information, e.g. do not disappear when this source of predictability is added.

## Results

The aim of our study was to examine the differential influence of different sources of predictability on dual-task performance in implicit and explicit learning groups. The results support the hypothesis of additive effects of prior knowledge and predictive visual information on dual-task tracking performance.

### Learning

A main effect of Segment in the Test Block demonstrated that learning of the repeating segment had taken place, *F*(3, 18) = 3.25, *p* = .046, η_p_^2^ = .351. Pairwise comparisons confirmed that performance on a random segment (*M* = 4.49, *SE* = 0.12) was worse than performance on the repeating segment placed on the left (*M* = 4.12, *SE* = 0.13), middle (*M* = 4.18, *SE* = 0.18) and on the right (*M* = 4.25, *SE* = 0.16; all *p*s < .05). This effect of Segment was similar for dual-task and single-task conditions, because there was no significant Segment × Condition interaction, *F*(3, 18) = 1.05, *p* = .396, η_p_^2^ = .149, and there was also no main effect of Condition, *F*(1, 20) = 4.20, *p* = .054, η_p_^2^ = .174. The effect of Segment also did not differ between the implicit group and explicit group, because no Segment × Group interaction was found, *F*(3, 18) < 1, *p* = .971, η_p_^2^ = .013. These were important to check since dual-task performance can suppress the expression of implicit knowledge (Cohen et al., 1990). Likewise, there was no Segment × Condition × Group interaction, *F*(3, 18) < 1, *p* = .462, η_p_^2^ = .130. To simplify the analysis of the learning effect, we took the repeating middle segments and the average of the random segments to create the two-level factor Segment (Repeating vs Random) in the analyses below. Given that we were mainly interested in the (additive) effects of predictability on dual-task performance, we will continue to report results on the individual contributions of each source of predictability on dual-task performance (first prior knowledge reflected on Segment, and then predictive visual information) in the Test and Retention Blocks. Results on single-task performance can be found in the supplementary material.

We also analyzed the implicit group’s answers on the questionnaire. From 10 participants, 9 indicated that they had not noticed a repeating segment (question 6) and also indicated the wrong segment in question 7. One participant indicated that she had noticed the repeating segment and crossed the middle one in question 7. Given that this participant’s values were not considerably lower than the other group’s participants and there was a 33% chance that the middle item was correct, the participant remained in the data set.

### Effects of Prior knowledge during dual tasking (Segment)

**Figure 4:**
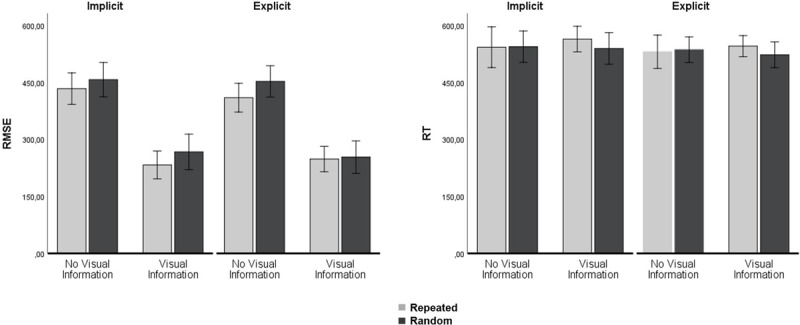
Test Block. Left: RMSE data showing dual-task tracking performance for the two groups. The combination of Predictive visual information and a Repeated segment (light grey bars) significantly improved tracking performance (additive effect). Right: Reaction Times for the two groups. There were no differences in RT, neither between random and repeated segments, nor for the two predictive visual information conditions. Error bars represent the standard error of the mean.

There was an effect of Segment for the Test Block, because tracking of repeating segments was better than tracking of random segments, *F*(1, 20) = 7.90, *p* = .011, η_p_^2^ = .283, see Figure 4. There was no significant Segment × Group interaction in the Test Block, F(1, 20) < 1, p = .796, η_p_^2^ = .003, showing that the effect was similar for the implicit and explicit learners.

**Figure 5:**
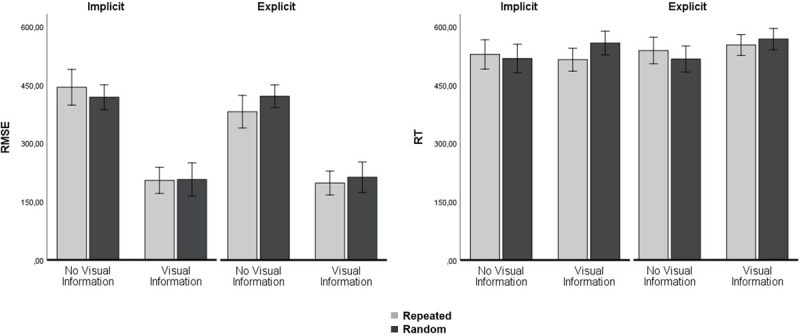
Retention Block. Left: RMSE data showing dual-task tracking performance for the two groups. The combination of Predictive visual information and a Repeated segment (light grey bars) significantly improved tracking performance (additive effect). Right: Reaction Times for the two groups. There were no differences in RT, neither between random and repeated segments, nor for the two predictive visual information conditions. Error bars represent the standard error of the mean.

There was no effect of Segment for the Retention Block, *F*(1, 20) = 3.05, *p* = .096, η_p_^2^ = .132, see Figure 5, but there was a significant Segment × Group effect, *F*(1, 20) = 6.48, *p* = .019, η_p_^2^ = .245, showing that only the explicit group benefited from the repeating segments in the Retention Block. In a separate ANOVA we tested whether the improvement in tracking from Test Block to Retention Block (comparing Figures 3 and 4) was significant, which was the case as indicated by a main effect of Block, *F*(1, 20) = 28.93, *p* < .001, η_p_^2^ = .591. Details show however that the specific improvement during repeated segments was more pronounced for the explicit group from Test to Retention Block, Segment × Group × Block *F*(1, 20) = 5.02, *p* = .037, η_p_^2^ = .201.

Reaction times were not significantly shorter while tracking a repeating segment, neither during the Test Block, *F*(1, 18) < 1, *p* = .377, η_p_^2^ = .044, nor during the Retention Block, *F*(1, 20) < 1, *p* = .388, η_p_^2^= .037.

### Effects of Predictive visual information during dual tasking

Predictive visual information significantly improved tracking during the Test Block, *F*(1, 20) = 238.69, *p* < .001, η_p_^2^ = .923, as well as during the Retention Block, *F*(1, 20) = 705.63, *p* < .001, η_p_^2^ = .972 (Figures 3 and 4). Predictive visual information did not significantly improve reaction times during the Test Block, *F*(1, 18) < 1, *p* = .726, η_p_^2^ = .007. However, in the Retention Block, there was a both a main effect of Predictive visual information on RT, *F*(1, 20) = 6.72, *p* = .017, η_p_^2^ = .251, and a significant Predictive visual information × Segment interaction, *F*(1, 20) = 9.97, *p* = .005, η_p_^2^ = .333 (Figure 5), because while predictive visual information had no effect on reaction times over the repeating segments it increased reaction times over the random segments (predictive information: *M* = 562 ms, *SE* = 10 ms vs. no predictive information: *M* = 517 ms, *SE* = 12 ms).

Exploratory analyses analyzing the first 5 trials when visual information was given showed that the benefit of visual information was instant, and did not develop over trials, as indicated by a non-significant main effect of Trial, *F*(1, 851), = 1.60, *p* = .206 and non-significant interactions between Trial, Visual Information, Segment and/or Block.

### Additive effects of prior knowledge (Segment) and predictive visual information during dual tasking

**Figure 6:**
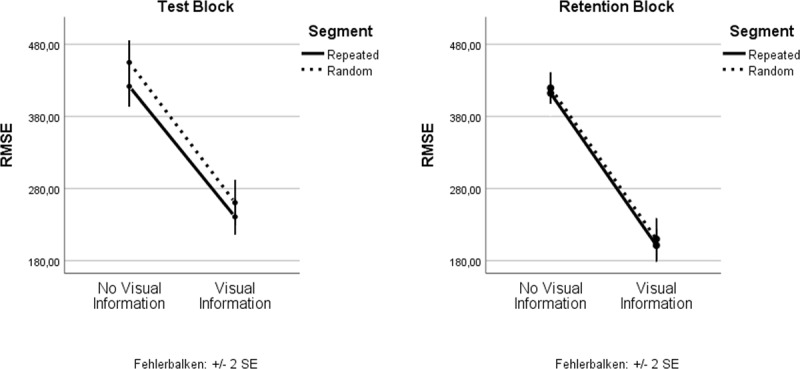
Additive effects of prior knowledge (Segment) and predictive visual information during dual tasking, showing that both sources of predictability aid dual tasking and that knowledge is not abandoned in favor of a feedforward control strategy.

If knowledge about the repeating segment and Predictive visual information were unconditional upon each other, there should be separate main effects and also a non-significant interaction. This was the case as shown by the main effects above, and the following non-significant Segment × Visual Information interaction in the Test Block, *F*(1, 20) < 1, *p* = .384, η_p_^2^ = .038, and the Retention Block, *F*(1, 20) < 1, *p* = .814, η_p_^2^ = .003. This shows that performance improvement in the repeating segment did not differ between trials with and without predictive visual information (see Figure 6).

Bayesian statistics using the Bayesian information criteria proposed by Wagenmakers (2007) were further applied to estimate the likelihood of the existence of the interaction (H_0_). In accordance Bayes factors classifications by van Doorn et al. (2019), the interaction term shows moderate evidence for H_0_, BF_10_ = .278.

## Discussion

The goal of the current study was to investigate the effect of prior knowledge and predictive visual information on dual-task performance. First, we needed to establish whether both sources of predictability influence dual-task performance. We found that predictive visual information had a strong impact on tracking performance and lowered tracking errors under dual-task conditions. The effects of prior knowledge were smaller and less consistent in the sense that implicit learning (of the repeated segment) was not demonstrable in all tests. However, learning in general was not redundant after adding visual information as we still found differences between random and repeated segments. We therefore conclude that both lead to increased performance in the presence of the other, confirming additive effects of predictability. This result extends research on the influence of predictability on dual-tasking which has been predominantly shown in experiments using discrete, sequentially-presented stimuli (Gaschler et al., 2018).

Our results showed no implicit learning effects at retention and two reasons may explain that: a ceiling effect in performance, or participants relying more on predictive visual information at the cost of implicit learning. The latter explanation is unlikely since the learning effect was absent when predictive visual information was unavailable. However, this explanation cannot be completely disregarded because it is possible that implicit knowledge faded out the more participants performed trials with predictive visual information which happened during the practice and retention blocks. Additionally, trials contained a repeating segment less consistently, or in a different position, during these blocks. While implicit knowledge is often retained better than explicit knowledge, it is also more susceptible to suffer when the task environment changes (Abrahamse et al., 2010; Lee & Vakoch, 1996). The existence of a ceiling effect is also possible because both groups improved significantly from the Practice to the Retention Block, although improvement stagnated on the repeating segment without predictive visual information. It must be noted that implicit learning effects in continuous task are more inconsistent than in SRT tasks. This has often been ascribed to methodological issues such as peculiarities of the repeating segment used (Chambaron et al., 2006; Van Ooteghem et al., 2008), but since the repeating segment was unique for every participant the implicit learning observed in the practice block is unlikely to be an anomaly. In contrast to the implicit group, we found the advantage of a repeating segment for the explicit group to be very consistent, this may be of little surprise since these participants were instructed regularly about the repeating segment. It is therefore difficult to establish how much the performance improvements were due to knowledge of the tracking path or a general direction of attention to the repeating segments through instructions.

Overall, the data from the Practice block suggest that participants were able to use information from prior knowledge and predictable visible information to optimize motor output. The effect of predictive visual information was much larger though, which may have had as a side effect that it was relied upon more with continued testing, as in the Retention Block. As described above, it is possible that this is the reason for implicit learning effects not showing in the Retention Block. Predictive visual information increased reaction times during the Retention Block although it is unclear why it happened only over the random segment. In previous work (Broeker et al., n.d.), we have manipulated predictability in the tracking task and the auditory task separately, and found that while visual information had no impact on RT, auditory sequences had no impact on RMSE, suggesting unilateral effects of predictability. If visual information serve visuomotor control, but audiomotor control requires auditory predictability, which was not manipulated in this study, then the fact that RTs increased for visually predictive and random segment speaks for this hypothesis. While the visual information was useless or even debilitative to reaction time performance, random segments increased processing load even further as they were never practiced or learnt/automatized. Beyond the assumption that predictability does not unconditionally reduce resource requirements, we assume that it may also add saliency to a task, which may channel more resources to that task despite instructions to pay equal attention to both tasks. A resource sharing theory of dual-task performance, where resources can be directed to tasks voluntarily or through task characteristics can best explain these findings (Tombu & Jolicœur, 2003; Wickens, 2008).

A limitation to the current study is that it could not be shown that participants reached a plateau in tracking performance by the end of training. Another Retention Block may have clarified whether implicit learning effects were due to a ceiling effect. In the original implicit learning tracking study by Pew (1974) learning of invariant features was not demonstrable until the sixth day of practice. However, the current training protocol was largely adopted from Ewolds et al. (2017), where learning was demonstrated after the same amount of trials, and some evidence pointed towards the bulk of the learning taking place during the first 20 trials.

It is important to investigate which factors may improve multitasking performance. A potential problem with adding information or informative cues to dual tasks is that they need to be processed under high sensory load. However, this study showed that different sources of predictability made available during dual-task do not hinder performance, but additively aid dual-task performance, at least for one dependent variable of interest. Future studies could take these results further by providing sources of predictability useful to both tasks, i.e. visuomotor and audiomotor control, to find further evidence that relevant cues and knowledge can help to circumvent processing limitations of the human system when performing multiple tasks.

## Data Accessibility Statement

Data of this project is available at https://osf.io/65hfk/.
